# The Risk of Obstructive Sleep Apnea Among Patients With Major Depressive Disorder and Generalized Anxiety Disorder in Saudi Arabia: A Cross-Sectional Study

**DOI:** 10.7759/cureus.101134

**Published:** 2026-01-09

**Authors:** Anas M Alswat, Sultan Qanash, Abdulaziz Aldahlawi, Abdulrahman Alhaddad, Asim Albishry, Sherin A Qari, Aseel S Jarwan, Alaa A Alkaki, Hani N Mufti, Badr Alaseeri, Hassan Alwafi, Abdallah Y Naser

**Affiliations:** 1 Medicine, King Saud Bin Abdulaziz University for Health Sciences, Jeddah, SAU; 2 Research, KAIMRC (King Abdullah International Medical Research Center), Jeddah, SAU; 3 Medicine, King Abdulaziz Medical City, Ministry of National Guard Health Affairs, Jeddah, SAU; 4 Cardiac Surgery, King Abdulaziz Medical City, Ministry of National Guard Health Affairs, Jeddah, SAU; 5 Pharmacology and Toxicology, Umm Al-Qura University, Mecca, SAU; 6 Applied Pharmaceutical Sciences and Clinical Pharmacy, Isra University, Faculty of Pharmacoepidemiology, Amman, JOR

**Keywords:** generalized anxiety disorder, major depression, obstructive sleep apnea, psychiatric disorder, sleep apnea, sleep quality

## Abstract

Introduction

Obstructive sleep apnea (OSA) is a common sleeping disorder characterized by recurring episodes of upper airway collapse during sleep. The relationship between OSA and psychiatric disorders, particularly depression and anxiety, has gained significant attention due to their frequent co-occurrence and bidirectional relationship. In this study, we assessed the risk of OSA among patients with psychiatric disorders and evaluated associated factors such as sleep quality and daytime sleepiness in the Saudi population.

Methods

This is a cross-sectional study that included patients with a confirmed diagnosis of major depressive and generalized anxiety disorders at King Abdulaziz Medical City in Jeddah, Saudi Arabia, from January 1 to December 31, 2023. Participants were interviewed in person and asked to fill out a survey that contains participants’ demographical data, validated Arabic versions of the Snoring, Tiredness, Observed apnea, high blood Pressure, BMI, Age, Neck circumference, Gender (STOP-BANG) questionnaire, Pittsburgh Sleep Quality Index (PSQI), Epworth Sleepiness Scale (ESS), Patient Health Questionnaire-9 (PHQ-9), and Generalized Anxiety Disorder-7 (GAD-7). Data was analyzed using appropriate statistical tests.

Results

A total of 183 patients diagnosed with major depressive disorder and/or generalized anxiety disorder were included. The study revealed that 27.9% (n=51) of participants were at high risk for OSA, scoring >3 on the STOP-BANG scale, with no significant difference between anxiety and depression diagnoses (p-value=0.737). Poor sleep quality was prevalent, with 73.2% (n=134) of participants scoring >5 on the PSQI scale. The median PSQI score was 8 (interquartile range (IQR)=5.00, 11.00), indicating poor overall sleep quality, with sleep latency, sleep efficiency, sleep disturbance, and daytime dysfunction components showing particular impairment. Anxiety patients demonstrated significantly higher daytime sleepiness compared to those with depression (p=0.041). In addition, we observed moderate to severe anxiety and depression in 26.8% (n=49) and 29% (n=53) of participants, respectively, highlighting a substantial psychiatric burden.

Conclusion

This study emphasizes the high prevalence of OSA risk and poor sleep quality among patients with psychiatric disorders. These findings underscore the need for comprehensive sleep evaluations in psychiatric populations and emphasize the importance of integrating sleep assessments into routine psychiatric care. Because of the complex bidirectional relationship between OSA and psychiatric disorders, tackling both conditions concurrently is crucial in clinical practice.

## Introduction

Obstructive sleep apnea (OSA) is characterized by periodic stops or reductions in airflow caused by a partial or total collapse of the upper airways despite exertion of respiratory effort [1]. OSA is associated with significant adverse health outcomes such as cardiovascular and metabolic illnesses when left untreated [2]. Nearly one billion people worldwide are affected by OSA, with the prevalence surpassing 50% in certain countries [3]. Up to 9% of middle-aged women and 25% of middle-aged men are affected by OSA [4]. Recently, in Saudi Arabia, it was found that over one-third of middle-aged Saudi men are at risk for OSA and require further evaluation to confirm or rule out the condition [4]. Furthermore, OSA appears to be more common among Saudi women [4]. Approximately 80% of adults with moderate to severe OSA are not recognized [2]. Complications of OSA include daytime sleepiness, reduced quality of life, decreased learning skills, and importantly, neurocognitive impairments that include impaired episodic memory, executive function, attention, and visuospatial cognitive functions. Such complications can have an influence on the patients’ quality of life and thus predispose them to psychiatric diseases such as anxiety and depression [5]. Multiple psychiatric disorders have been connected to OSA and can negatively impact treatment and worsen the prognosis [6].

Previous studies have shown a positive correlation between OSA and psychiatric illnesses such as anxiety and depression in that the presence of a psychiatric comorbidity with OSA can affect the patient’s compliance with treatment and quality of life [6]. Additionally, it has been demonstrated that mood disorders are prevalent in OSA patients, with their incidence tending to rise with OSA severity and fall in those on continuous positive airway pressure [6]. Previous studies found that individuals diagnosed with psychiatric illness and suspected OSA experience psychiatric symptoms that are more severe than regular sleepers [7,8]. It has also been proven that comorbid OSA in individuals with major depressive disorder induces increased suicidality and worse outcomes [7].

The association between OSA and psychiatric disorders has been established. However, further research is required to assess the association strength and to discover the factors that play a role in this correlation [8]. Also, further inquiries need to be conducted to address the impact of psychotropic medications on OSA symptoms and choice of pharmacotherapy in patients with OSA [9,10]. In addition, psychiatric patients experience symptoms that are consistent with OSA symptoms, such as sleepiness during the day, concentration problems, insomnia, fatigue, and low energy, but its correlation with OSA is not addressed [9].

Therefore, this study aimed to assess the risk of OSA in patients with psychiatric disorders, particularly major depressive and generalized anxiety disorders, and its association with patients’ demographics. Also, we aimed to evaluate the quality of sleep and the degree of daytime sleepiness in patients with these psychiatric disorders.

## Materials and methods

Study design and participants

A cross-sectional study was carried out at King Abdulaziz Medical City in Jeddah, Saudi Arabia, from January 2023 to December 2023. We included patients who are more than 18 years old with a confirmed diagnosis of major depressive disorder and/or generalized anxiety disorder by a psychiatrist. We excluded those who have already been diagnosed with OSA or any other sleep disorders. To maximize the quality of data obtained, patients who attended the psychiatry clinic were interviewed in person and given a survey to be filled out. Informed consent was obtained.

Study measures

Participants’ characteristics such as age, BMI, marital status, employment status, comorbidities (e.g., diabetes, hypertension, dyslipidemia, chronic kidney disease, etc.), current diagnosis of psychiatric disorders (e.g., generalized anxiety disorder, major depression, bipolar disorder, schizophrenia, obsessive compulsive disorder, etc.), and use of any psychiatric medications selective serotonin reuptake inhibitors (SSRIs), serotonin-norepinephrine reuptake inhibitors (SNRIs), tricyclic antidepressants (TCAs), anxiolytics, etc.) were collected. One component of the survey was an Arabic-validated version of the Snoring, Tiredness, Observed apnea, high blood Pressure, BMI, Age, Neck circumference, Gender (STOP-BANG) sleep apnea questionnaire [11]. The STOP-BANG questionnaire is used as a screening tool that assesses eight risk factors for obstructive sleep apnea (OSA) [12]. Scores range from 0 to 8, with a score ≥3 indicating high risk for OSA. Particularly for moderate to severe OSA, the Arabic version has shown high sensitivity and negative predictive value [11].

Another part of the survey was an Arabic validated version of the Pittsburgh Sleep Quality Index (PSQI) [13,14]. PSQI is a self-report scale measuring sleep quality over a one-month period, with scores ranging from 0 to 21 [15]. A global PSQI score >5 indicates poor sleep quality. The Arabic translation of the PSQI has demonstrated excellent validity of construct and acceptable internal consistency.

To assess for daytime sleepiness, an Arabic version of the Epworth Sleepiness Scale (ESS) was used [16]. ESS is an eight-item questionnaire measuring daytime sleepiness, with scores ranging from 0 to 24. While a cut-off of ≥10 is commonly used to indicate excessive daytime sleepiness, recent research suggests an optimal cut-off of 16 [17]. The Arabic version of the ESS (ArESS) has been validated as a reliable tool for measuring daytime sleepiness in Arabic-speaking populations [16].

For measurement of psychiatric burden, we used the Patient Health Questionnaire-9 (PHQ-9) and Generalized Anxiety Disorder-7 (GAD-7) for assessment of depression and anxiety, respectively [18-21]. PHQ-9 is a depression screening tool with scores ranging from 0 to 27 [22]. Cut-off scores of 8 to 11 have been proven to have good diagnostic qualities for recognizing major depressive disorder. GAD-7 is a brief measure for assessing anxiety, with scores ranging from 0 to 21, with mild, moderate, and severe levels of anxiety being represented by 5, 10, and 15 cut-off points, respectively [23].

Statistical analysis

The data were analyzed using R statistical package software (R Foundation for Statistical Computing, Vienna, Austria) and/or Python (Python Software Foundation (PSF), Wilmington, Delaware, USA). Because most of the continuous variables in our dataset were not normally distributed, we chose to use the median and interquartile range (IQR). To investigate whether a significant difference existed between patients with the outcome of interest, the Wilcoxon rank sum test was utilized. Frequencies and percentages representing categorical variables were provided, and the appropriate Chi-square or Fisher's exact test was used for analysis. Ordinal attributes were analyzed by the Kruskal-Wallis test. Every statistical test was two-tailed. The 95% CI and a p value <0.05 were used for statistical significance. The study was conducted and approved by the Institutional Review Board of King Abdullah International Medical Research Center, Jeddah, Saudi Arabia (Approval number: IRB/2153/22, approved on October 11, 2022).

## Results

Participants demographics

The final analysis of this study included a total of 183 participants. Our data demonstrates that the median age of the participants was 37 years (IQR=28.50, 49.50); 114 were females (62.3%), and the majority were married (60.1%). The median weight was 75.28 kg (IQR=65.00, 83.50), with a median BMI of 27.80 (IQR=24.76, 31.24), indicating that most participants were overweight. The most common psychiatric diagnoses were depression (52.5%) and anxiety (15.8%). Most of the participants (87.4%) were not previously diagnosed with any sleep disorder, while 12.6% were diagnosed with insomnia. Additionally, 34.4% of participants were using psychiatric medication, while 65% were not. More demographic data are shown in Table 1.

**Table 1:** Demographic characteristics of participants (n=183).

	Level	Overall	Anxiety	Depression	Depression and Anxiety	Others	p
n		183	30	101	31	21	
Age in years (median (IQR))		37.00 (28.50, 49.50)	44.00 (30.75, 52.75)	33.00 (26.00, 49.00)	38.00 (30.00, 48.50)	36.00 (30.00, 47.00)	0.232
Gender, n (%)	Female	114 (62.3)	20 (66.7)	62 (61.4)	21 (67.7)	11 (52.4)	0.671
	Male	69 (37.7)	10 (33.3)	39 (38.6)	10 (32.3)	10 (47.6)	
Weight in kg (median (IQR))		75.00 (65.00, 83.50)	66.50 (60.25, 83.00)	75.00 (66.00, 81.00)	76.00 (62.50, 88.50)	80.00 (69.00, 85.00)	0.512
Height in m (median (IQR))		1.62 (1.57, 1.69)	1.64 (1.59, 1.66)	1.60 (1.56, 1.70)	1.61 (1.57, 1.68)	1.65 (1.58, 1.71)	0.821
BMI (median (IQR))		27.80 (24.76, 31.24)	25.65 (23.29, 29.94)	27.95 (25.20, 30.49)	27.56 (24.43, 34.33)	30.12 (25.65, 32.34)	0.228
Marital status (%)	Divorced	10 (5.5)	1 (3.3)	7 (6.9)	2 (6.5)	0 (0.0)	0.361
	Married	110 (60.1)	19 (63.3)	53 (52.5)	23 (74.2)	15 (71.4)	
	Single	53 (29.0)	7 (23.3)	35 (34.7)	6 (19.4)	5 (23.8)	
	Widowed	10 (5.5)	3 (10.0)	6 (5.9)	0 (0.0)	1 (4.8)	
Employment (%)	Employed	74 (40.4)	16 (53.3)	40 (39.6)	11 (35.5)	7 (33.3)	0.605
	Not-employed	71 (38.8)	9 (30.0)	40 (39.6)	11 (35.5)	11 (52.4)	
	Retired	19 (10.4)	2 (6.7)	9 (8.9)	6 (19.4)	2 (9.5)	
	Student	19 (10.4)	3 (10.0)	12 (11.9)	3 (9.7)	1 (4.8)	
Monthly income (%)	<5K	81 (44.3)	12 (40.0)	42 (41.6)	16 (51.6)	11 (52.4)	0.002
	>15K	15 (8.2)	9 (30.0)	4 (4.0)	1 (3.2)	1 (4.8)	
	10-15K	25 (13.7)	4 (13.3)	15 (14.9)	3 (9.7)	3 (14.3)	
	5-10K	62 (33.9)	5 (16.7)	40 (39.6)	11 (35.5)	6 (28.6)	
Smoker (%)	Ex	5 (2.7)	2 (6.7)	2 (2.0)	1 (3.2)	0 (0.0)	0.342
	N	129 (70.5)	19 (63.3)	69 (68.3)	22 (71.0)	19 (90.5)	
	Y	49 (26.8)	9 (30.0)	30 (29.7)	8 (25.8)	2 (9.5)	

The risk of OSA and its correlation with psychiatric disorders

Of all participants, 72.1% (n=132) have a STOP-BANG score of <3, indicating a low risk for obstructive sleep apnea. On the other hand, a considerable portion of participants, 27.9% (n=51), were at high risk for obstructive sleep apnea, scoring >3 in the STOP-BANG score. There was no statistically significant difference between participants diagnosed with anxiety or depression in terms of increased risk of OSA (p-value=0.737). More details are given in Table 2.

**Table 2:** STOP-BANG scores. STOP-BANG: Snoring, Tiredness, Observed apnea, high blood Pressure, BMI, Age, Neck circumference, Gender.

	Level	Overall	Anxiety	Depression	Depression and Anxiety	Others	p
n		183	30	101	31	21	0.737
STOP-BANG score (%)	Low risk	132 (72.1)	20 (66.7)	76 (75.2)	22 (71.0)	14 (66.7)	
	High risk	51 (27.9)	10 (33.3)	25 (24.8)	9 (29.0)	7 (33.3)	

PSQI and ESS scale scores

The analysis showed that the median Pittsburgh Sleep Quality Index score was 8, indicating poor sleep quality on average among all participants. Most participants, 73.2% (n=134), had poor sleep quality, scoring >5 on the PSQI scale, while only 26.8% (n=49) had good sleep quality, scoring <5 on the scale. No statistically significant difference was found between participants diagnosed with anxiety or depression (p-value=0.608), and the global PSQI score was high, indicating poor sleep quality in most of the participants, with most of the components being met in the PSQI scale. Additionally, the Subjective Sleep Quality component of the PSQI had a median score of 1, demonstrating good sleep quality. However, an analysis of the sleep latency, sleep efficiency, sleep disturbance, and daytime dysfunction components of the PSQI score showed a median score of 2 or higher, indicating poor sleep quality in these areas (Table 3).

**Table 3:** PSQI and ESS scores. From Ben Letaifa et al. [13], Buysse et al. [14], Beaudreau et al. [15], Ahmed et al. [16], and Lapin et al. [17]. PSQI: Pittsburgh Sleep Quality Index, ESS: Epworth Sleepiness Scale.

	Level	Overall	Anxiety	Depression	Depression and Anxiety	Others	p
n		183	3	101	31	21	
Pittsburgh Sleep Quality Index (median (IQR))		8.00 (5.00, 11.00)	9.00 (6.25, 11.00)	8.00 (5.00, 10.00)	8.00 (6.00, 11.50)	7.00 (4.00, 11.00)	0.478
Pittsburgh Sleep Quality Index, severity level (%)	Good sleep quality	49 (26.8)	7 (23.3)	27 (26.7)	7 (22.6)	8 (38.1)	0.608
	Poor sleep quality	134 (73.2)	23 (76.7)	74 (73.3)	24 (77.4)	13 (61.9)	
	High risk	51 (27.9)	10 (33.3)	25 (24.8)	9 (29.0)	7 (33.3)	
ESS score (median (IQR))		4.00 (0.00, 8.00)	6.50 (0.00, 9.75)	3.00 (0.00, 5.00)	3.00 (0.50, 9.00)	5.00 (0.00, 8.00)	0.251
ESS score, severity level (%)	Low daytime sleepiness	121 (66.1)	14 (46.7)	76 (75.2)	18 (58.1)	13 (61.9)	0.041
	High daytime sleepiness	37 (20.2)	9 (30.0)	17 (16.8)	7 (22.6)	4 (19.0)	
	Mild excessive daytime sleepiness	13 (7.1)	5 (16.7)	5 (5.0)	3 (9.7)	0 (0.0)	
	Moderate excessive daytime sleepiness	7 (3.8)	1 (3.3)	3 (3.0)	1 (3.2)	2 (9.5)	
	Severe excessive daytime sleepiness	5 (2.7)	1 (3.3)	0 (0.0)	2 (6.5)	2 (9.5)	

The median Epworth Sleepiness Scale score was 4, indicating mild daytime sleepiness. The majority of participants, 66.1% (n=121), had low daytime sleepiness, while 20.2% (n=37) had high daytime sleepiness. Additionally, 7.1%, 3.8%, and 2.7% of the participants have mild, moderate, and severe excessive daytime sleepiness, respectively. Participants with anxiety have higher scores in ESS compared to those with depression, indicating increased daytime sleepiness, and this difference was statistically significant (p-value=0.041). Additional details are given in Table 3.

GAD-7 and PHQ-9 scores

The median GAD-7 score was 5, indicating mild anxiety. The majority of participants (45.9%) had minimal anxiety, while 26.8 % (n=49) had moderate to severe anxiety, scoring >10 points on the scale. The median PHQ-9 score was 5, indicating mild depression. The majority of participants (43.2%) had minimal depression, while 29 % (n=53) had moderate, moderately severe, and severe depression, scoring >10 points on the PHQ-9 scale. Table 4 shows additional details.

**Table 4:** PHQ-9 and GAD-7 scores. From AlHadi et al. [18], Snijkers et al. [19], Spitzer et al. [20], Kroenke and Spitzer [21], Summaka et al. [22], and Patrick and Connick [23]. PHQ-9: Patient Health Questionnaire-9, GAD-7: Generalized Anxiety Disorder-7.

	Level	Overall	Anxiety	Depression	Depression and Anxiety	Others	p
n		183	30	101	31	21	
GAD-7 score (median (IQR))		5.00 (2.00, 10.50)	8.00 (5.25, 10.75)	3.00 (1.00, 7.00)	9.00 (5.00, 14.00)	3.00 (0.00, 12.00)	<0.001
GAD-7 score anxiety severity level (%)	Minimal anxiety	84 (45.9)	7 (23.3)	60 (59.4)	6 (19.4)	11 (52.4)	0.001
	Mild anxiety	50 (27.3)	13 (43.3)	23 (22.8)	11 (35.5)	3 (14.3)	
	Moderate anxiety	28 (15.3)	6 (20.0)	8 (7.9)	9 (29.0)	5 (23.8)	
	Severe anxiety	21 (11.5)	4 (13.3)	10 (9.9)	5 (16.1)	2 (9.5)	
PHQ-9 questionnaire score (median (IQR))		5.00 (3.00, 11.00)	6.50 (2.00, 11.00)	5.00 (3.00, 8.00)	9.00 (3.00, 15.00)	6.00 (2.00, 10.00)	0.207
PHQ-9 questionnaire score depression severity level (%)	Minimal depression	79 (43.2)	12 (40.0)	45 (44.6)	12 (38.7)	10 (47.6)	0.024
	Mild depression	51 (27.9)	8 (26.7)	35 (34.7)	4 (12.9)	4 (19.0)	
	Moderate depression	19 (10.4)	5 (16.7)	4 (4.0)	5 (16.1)	5 (23.8)	
	Moderately severe depression	19 (10.4)	5 (16.7)	8 (7.9)	5 (16.1)	1 (4.8)	
	Severe depression	15 (8.2)	0 (0.0)	9 (8.9)	5 (16.1)	1 (4.8)	

## Discussion

OSA is a common sleep disease that involves recurring bouts of upper airway collapse during sleep, resulting in intermittent hypoxia and sleep fragmentation. The relationship between OSA and psychiatric disorders, particularly depression and anxiety, has gained significant attention in recent years due to their frequent co-occurrence and potential bidirectional relationship. In this study, we tried to assess the risk of OSA among patients diagnosed with psychiatric illnesses, particularly anxiety and depression, and also identify associated factors like patients’ demographics, quality of sleep, and daytime sleepiness.

The pathophysiological mechanisms behind the connection between OSA and psychiatric disorders are multifaceted and involve several key processes. One of the proposed mechanisms is hypoperfusion, a consequence of recurrent apneic episodes, which leads to reduced cerebral blood flow and oxygen delivery, potentially affecting brain regions crucial for mood regulation and cognitive function [24,25]. In addition, neuroinflammation, triggered by intermittent hypoxia and sleep fragmentation, can also disrupt neurotransmitter systems and contribute to the development or exacerbation of psychiatric symptoms [25,26]. Moreover, endothelial dysfunction, a hallmark of OSA, may compromise the blood-brain barrier integrity, further contributing to neuroinflammation and potentially increasing vulnerability to psychiatric disorders [27]. These interconnected processes create a vicious cycle that may explain the high comorbidity between OSA and psychiatric conditions, as well as the potential for OSA to serve as an independent risk factor for developing psychiatric illnesses [26,27].

Our results revealed that 27.9% of participants were at high risk for OSA, as indicated by a STOP-BANG score ≥3. Interestingly, we found no statistically significant difference in OSA risk between participants diagnosed with anxiety or depression. The majority of participants (73.2%) exhibited poor sleep quality, as measured by the Pittsburgh Sleep Quality Index (PSQI), with a median score of 8. This poor sleep quality was consistent across anxiety and depression diagnoses. Further analysis of the PSQI components revealed that sleep latency, sleep efficiency, sleep disturbance, and daytime dysfunction had median scores of 2 or higher, indicating specific areas of poor sleep quality. Notably, participants with anxiety demonstrated significantly higher daytime sleepiness, as measured by the Epworth Sleepiness Scale (ESS), compared to those with depression. The GAD-7 and PHQ-9 scores indicated that a substantial proportion of participants experienced moderate to severe anxiety (26.8%) and depression (29%), highlighting the significant psychiatric burden in our sample.

The findings in our study are largely consistent with existing literature, although some differences are noteworthy. The prevalence of high OSA risk in our study (27.9%) is comparable to estimates in the general population. A systematic review found that OSA prevalence at Apnea-Hypopnea Index (AHI) ≥5 events/h ranged from 9% to 38% in the general adult population; however, polysomnography was used to assess OSA in this systematic review [6]. Additionally, a French study found a total weighted prevalence of treated sleep apnea or high risk of OSA of 20.9% [28]. This study used the Berlin Questionnaire for assessing OSA risk [29]. On the other hand, our results contrast with some studies that have reported higher OSA prevalence in psychiatric populations. For instance, the study by Gupta and Simpson (2015) found that up to 50% of patients with major depressive disorder had comorbid OSA [6]. The lack of significant difference in OSA risk between anxiety and depression diagnoses in our study is intriguing and warrants further investigation. Another study by Shoib et al. (2022) found that 39.5% of psychiatric inpatients were at high risk for OSA, with depressive symptoms increasing the likelihood of OSA risk by four times [30]. Additionally, a large cross-sectional study of over 18,000 individuals from five countries reported that individuals with depression were five times more likely to have a breathing-related sleep problem, even after accounting for other OSA-related factors [31].

Sleep quality plays a crucial role, as poor sleep quality in psychiatric patients with OSA exacerbates psychiatric symptoms and impairs treatment response [32]. Our results were comparable to the literature in terms of the association between psychiatric disorders and poor sleep. The high prevalence of poor sleep quality (73.2%) in our sample exceeds typical rates in the general population, which range from 10% to 30% [6]. This underscores the significant impact of psychiatric disorders on sleep patterns. The higher daytime sleepiness observed in anxiety patients compared to those with depression is an interesting finding that adds to the growing body of evidence suggesting distinct sleep-related manifestations across different psychiatric conditions. Furthermore, other researches also discovered that OSA patients who reported insomnia-like symptoms or sleepiness did not necessarily exhibit a more severe OSA phenotype, but their subjective sleep quality was substantially worse, possibly leading to a vicious cycle of depression and anxiety [31,33].

Our study has some limitations that should be considered. First, because the study was cross-sectional, causal relationships between psychiatric disorders and sleep disturbances could not be established. Second, relying on self-reported measures may, in part, result in reporting bias. Future research should incorporate objective sleep measures, such as polysomnography or actigraphy, to complement subjective assessments. Additionally, longitudinal studies are needed to elucidate the temporal relationship between psychiatric disorders and sleep disturbances, including OSA risk. Investigating the impact of OSA treatment on psychiatric symptoms and exploring potential biomarkers for early detection of OSA in psychiatric populations could provide valuable insights for clinical practice.

## Conclusions

In conclusion, our study highlights the considerable risk of OSA and the high prevalence of poor sleep quality among patients with psychiatric disorders, particularly depression and anxiety. While the OSA risk was not significantly different between anxiety and depression diagnoses, the overall poor sleep quality and increased daytime sleepiness, especially in anxiety patients, underscore the need for comprehensive sleep evaluations in psychiatric populations. Future research should focus on elucidating the complex bidirectional relationships between OSA and psychiatric disorders to inform targeted interventions and treatment strategies.
